# (*E*)-2-[(2-Hydr­oxy-5-nitro­phen­yl)iminiometh­yl]-4-nitro­phenolate

**DOI:** 10.1107/S1600536809000543

**Published:** 2009-01-14

**Authors:** Yousef M. Hijji, Belygona Barare, Ray J. Butcher, Jerry P. Jasinski

**Affiliations:** a Department of Chemistry, Morgan State University, Baltimore, MD 21251, USA; b Department of Chemistry, Howard University, 525 College Street NW, Washington DC 20059, USA; c Department of Chemistry, Keene State College, 229 Main Street, Keene, NH 03435-2001, USA

## Abstract

The title mol­ecule, C_13_H_9_N_3_O_6_, consists of a 2-hydr­oxy-5-nitro­phenyl­iminio group and a 4-nitro­phenolate group bonded to a methyl­ene C atom with both of the planar six-membered rings nearly in the plane of the mol­ecule [dihedral angle = 1.3 (4)°]. Each of the nitro O atoms is twisted slightly out of the plane of the mol­ecule. The amine group forms an intra­molecular hydrogen bond with both nearby O atoms, each of which has partial occupancy of attached H atoms [0.36 (3) and 0.64 (3)]. An extended π-delocalization throughout the entire mol­ecule exists producing a zwitterionic effect in this region of the mol­ecule. The shortened phenolate C—O bond [1.2749 (19)°], in concert with the slightly longer phenol C—O bond [1.3316 (19) Å], provides evidence for this effect. The crystal packing is influenced by extensive strong inter­molecular O—H⋯O hydrogen bonding between the depicted phenolate and hydr­oxy O atoms and their respective H atoms within the π-delocalized region of the mol­ecule. As a result, mol­ecules are linked into an infinite polymeric chain diagonally along the [110] plane of the unit cell in an alternate inverted pattern. A MOPAC AM1 calculation provides support for these observations.

## Related literature

For related structures, see: Butcher *et al.* (2007 ▶); Ersanlı *et al.* (2003 ▶); Gül *et al.* (2007 ▶); Hijji *et al.* (2008 ▶); Odabaşoğlu *et al.* (2006 ▶); Jasinski *et al.* (2007 ▶). For related literature, see: Schmidt & Polik (2007 ▶).
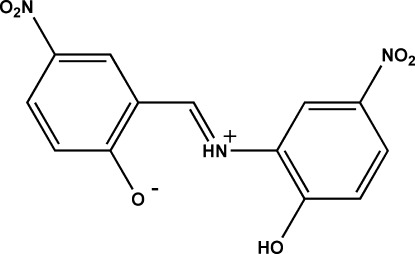


## Experimental

### 

#### Crystal data

C_13_H_9_N_3_O_6_
*M* *_r_* = 303.23
Monoclinic, 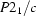

*a* = 7.9649 (1) Å
*b* = 8.6110 (1) Å
*c* = 19.1190 (3) Å
β = 98.433 (2)°
*V* = 1297.11 (3) Å^3^
*Z* = 4
Mo *K*α radiation
μ = 0.13 mm^−1^
*T* = 296 (2) K
0.37 × 0.27 × 0.18 mm

#### Data collection

Oxford Diffraction Gemini R diffractometer
Absorption correction: multi-scan (*CrysAlis RED*; Oxford Diffraction, 2007 ▶) *T*_min_ = 0.954, *T*_max_ = 0.978
6432 measured reflections
2495 independent reflections
1819 reflections with *I* > 2σ(*I*)
*R*_int_ = 0.023

#### Refinement

*R*[*F* ^2^ > 2σ(*F* ^2^)] = 0.041
*wR*(*F* ^2^) = 0.129
*S* = 1.05
2495 reflections
202 parameters
H-atom parameters constrained
Δρ_max_ = 0.22 e Å^−3^
Δρ_min_ = −0.17 e Å^−3^

### 

Data collection: *CrysAlisPro* (Oxford Diffraction, 2007 ▶); cell refinement: *CrysAlisPro*; data reduction: *CrysAlisPro* program(s) used to solve structure: *SHELXS97* (Sheldrick, 2008 ▶); program(s) used to refine structure: *SHELXL97* (Sheldrick, 2008 ▶); molecular graphics: *SHELXTL* (Sheldrick, 2008 ▶); software used to prepare material for publication: *SHELXTL*.

## Supplementary Material

Crystal structure: contains datablocks global, I. DOI: 10.1107/S1600536809000543/hg2463sup1.cif

Structure factors: contains datablocks I. DOI: 10.1107/S1600536809000543/hg2463Isup2.hkl

Additional supplementary materials:  crystallographic information; 3D view; checkCIF report

## Figures and Tables

**Table 1:** Hydrogen-bond geometry (Å, °)

*D*—H⋯*A*	*D*—H	H⋯*A*	*D*⋯*A*	*D*—H⋯*A*
O1—H1*O*⋯O2^i^	0.82	1.77	2.5570 (16)	161
O2—H2*O*⋯O1^ii^	0.82	1.75	2.5570 (16)	166
N1—H1*N*⋯O1	0.86	1.90	2.6001 (19)	138
C3—H3*A*⋯O3^iii^	0.93	2.56	3.295 (2)	137
C7—H7*A*⋯O4^iv^	0.93	2.67	3.289 (2)	125
C7—H7*A*⋯O5^v^	0.93	2.44	3.312 (2)	156
C10—H10*A*⋯O4^vi^	0.93	2.53	3.321 (2)	143
C13—H13*A*⋯O4^iv^	0.93	2.64	3.195 (2)	119
C13—H13*A*⋯O5^v^	0.93	2.63	3.512 (2)	160

Symmetry codes: (i) 
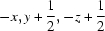
; (ii) 
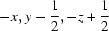
; (iii) 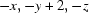; (iv) 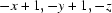; (v) 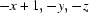; (vi) 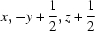.

**Fig. 1.**
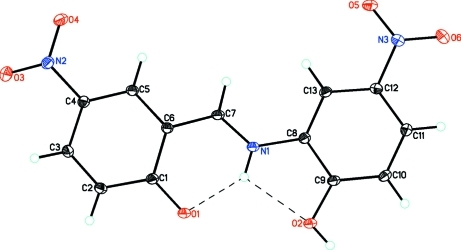
The molecular structure of C13H9N3O6, showing the atom numbering scheme and 50% probability displacement ellipsoids. H1O (0.36 (3) occupancy) has been omitted from O1 in the diagram and only the predominant component, H2O (0.64 (3) occupancy), has been shown. Dashed lines indicate intramolecular N–H···O hydrogen bonds.

**Fig. 2.**
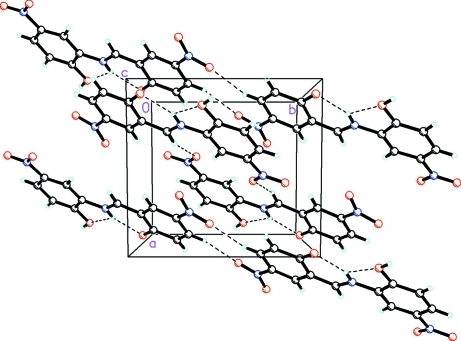
The molecular packing for C13H9N3O6 viewed down the c axis. Dashed lines indicate intermolecular O–H···O, C–H···O and intramolecular N–H···O hydrogen bonds. The predominately occupied (0.64) hydrogen atom (H2O) is shown attached to O2 while H1O at 0.36 occupancy is not depicted.

## supplementary crystallographic information

### Comment

Schiff bases have a wide range of application in chemistry. The title compound, a Schiff base derivative, was synthesized under microwave conditions and recrystallized from ethanol to give brown crystals. The structural data shows that it exists as an iminio-phenolate zwitterion in the solid state. Typically, keto-amine tautomer behavior has been observed in related derivative compounds (Butcher *et al.* (2007); Jasinski *et al.* (2007); Gül *et al.* (2007); Odabaşoğlu *et al.* (2006)); Ersanlı *et al.* (2003)). Compounds of this type can be used as anion sensors in acetonitrile (Hijji *et al.* (2008)) that tend to exist in the keto-amine form, which is generally favored over the phenol-imine form in the solid state. Introduction of electron deficient groups to the aromatic rings tends to increase the acidity of the phenolic proton.

The title molecule, C_13_H_9_N_3_O_6_, consists of a 2-hydroxy-5-nitrophenyliminio group and a 4-nitrophenolate group bonded to a methylene carbon atom with both of the planar six-membered rings nearly in the plane of the molecule. The dihedral angle between the mean planes of the phenyl and phenolate rings measures 1.3 (4)°. Each of the nitro oxygen atoms are twisted slightly out of the plane of the molecule [torsion angles = 172.16 (17)° (O3—N2—C4—C5); -7.1 (2)° (O4—N2—C4—C5); -7.6 (3)° (O3—N2—C4—C3); 173.17 (15)° (O4—N2—C4—C3); and 178.63 (16)° (O6—N3—C12—C13); -3.4 (3)° (O5—N3—C12—C13); 174.28 (18)° (O5—N3—C12—C11); -3.7 (3)° (O6—N3—C12—C11)]. The phenolate (O1) and hydroxy (O2) oxygen atoms are essentially in the plane of the molecule [torsion angles = 179.03 (16)° (O1—C1—C6—C5); 0.5 (3)° (O1—C1—C6—C7); 179.31 (18)° (O2—C9—C10—C11); -177.90 (15)° (C13—C8—C9—O2)]. The imino group forms an intramolecular hydrogen bond with each of the nearby oxygen atoms (O1 and O2) which have partial occupancy of hydrogen atoms (H1O [0.36 (3)] and H2O [0.64 (3)], respectively) (see Fig. 1 which shows only the predominant component, H2O, and Table 2). There appears to be an extended π delocalization effect throughout the entire molecule producing a zwitterionic effect in this region of the molecule. The shortened C1—O1 bond (1.2749 (19) Å in concert with the slightly longer C9—O2 bond (1.3316 (19) Å) provide structural evidence for this effect.

Crystal packing is influenced by extensive strong intermolecular O—H···O hydrogen bonding between the depicted phenolate and hydroxy oxygen atoms (O1 & O2) and their respective hydrogen atoms within the π delocalized region (O1—H1O(0.36)···O2; 2.5570 (16) Å) and O2—H2O(0.64)···O1; 2.5570 (16) Å) of the molecule. Additional weak intermolecular C—H···O hydrogen bond interactions occur involving the methylene carbon (C7) and the phenyl (C10 & C13) and phenolate (C3) groups (Fig. 2), respectively. All of the hydrogen bond interactions are summarized in Table 1. As a result the molecules are linked into an infinite polymeric chain diagonally along the [110] plane of the unit cell in an alternate inverted pattern (Fig. 2). In addition, weak *Cg*1–*Cg*1 (3.517 (2) Å; slippage = 1.09 (8)°; *-x, 1 - y, -z*) and *Cg*1–*Cg*2 (3.830 (6) Å; *x, y - 1, z*) π-π stacking ring interactions also occur where *Cg*1 = center of gravity of the C1–C6 ring and *Cg*2 = center of gravity of the C8–C13 ring.

After a *MOPAC* AMI calculation [Austin Model 1 approximation together with the Hartree-Fock closed-shell (restricted) wavefunction was used and minimizations were terminated at an r.m.s. gradient of less than 0.01 kJ mol^-1^ Å^-1^] of the zwitterionic form with *WebMO Pro* (Schmidt, 2007). As a result of this energy minimization, the dihedral angle between the phenyl and phenolate rings changes from 1.3 (4)° in the crystal structure to 7.6 (6)°, producing a slightly more twisted molecule than the nearly planar molecule in the crystalline environment. Thus, it is apparent that the extensive hydrogen bonding and π-π stacking intermolecular interactions significantly influence crystal packing with this molecule.

### Experimental

The title compound was synthesized as follows: 2-amino-4-nitrophenol (0.15 g, 1 mmol) and 2-hydroxy-5-nitrobenzaldehyde (0.17 g, 1 mmol) were mixed in a loosely capped vial. The reaction mixture was allowed to heat at full power in a conventional microwave for 8 minutes. The compound was recrystallized from ethanol affording a brown solid (0.20 g, 68%). (mp 591–593 K) ^1^H-NMR (400 MHz, DMSO-d_6_), δ (p.p.m.): 14.64 (s, br, 1H), 11.74 (s, br, 1H) 9.37 (s, 1H), 8.72 (d, J = 2.87 Hz, 1 H), 8.41 (d, J = 3.3 Hz, 1H), 8..27 (dd, J = 9.2, 3.1 Hz, 1H), 8.13 (1*H*, dd, J = 9.06, 2.7 Hz, 1 H), 7.16 (d, J = 9.1 Hz, 1H), 7.09 (d, J = 9.2 Hz, 1H), ^13^C-NMR (100 MHz, DMSO-d_6_) δ (p.p.m.): 167.90, 162.13, 157.48, 139.89, 138.76, 133.40, 128.95, 128.69, 124.51, 118.78, 118.25, 116.54, 115.24.

### Refinement

H1A, H1O and H2O were obtained from a difference Fourier map. The occupancies of H1O and H2O refined to values of 0.36 (3) and 0.64 (3), respectively. The rest of the H atoms were placed in their calculated positions and then refined using the riding model with C(N,*O*)—H = 0.82 to 0.93 Å, and with *U*_iso_(H) = 1.15–1.20*U*_eq_(C,*N*).

### Crystal data

**Table:**

C_13_H_9_N_3_O_6_	*F*(000) = 624
*M**_r_* = 303.23	*D*_x_ = 1.553 Mg m^−^^3^
Monoclinic, *P*2_1_/*c*	Mo *K*α radiation, λ = 0.71073 Å
Hall symbol: -P 2ybc	Cell parameters from 3209 reflections
*a* = 7.9649 (1) Å	θ = 3.9–73.2°
*b* = 8.6110 (1) Å	µ = 0.13 mm^−^^1^
*c* = 19.1190 (3) Å	*T* = 296 K
β = 98.433 (2)°	Prism, orange-brown
*V* = 1297.11 (3) Å^3^	0.37 × 0.27 × 0.18 mm
*Z* = 4	

### Data collection

**Table:**

Oxford Diffraction Gemini R diffractometer	2495 independent reflections
Radiation source: fine-focus sealed tube	1819 reflections with *I* > 2σ(*I*)
graphite	*R*_int_ = 0.023
Detector resolution: 10.5081 pixels mm^-1^	θ_max_ = 26.2°, θ_min_ = 2.6°
φ and ω scans	*h* = −9→9
Absorption correction: multi-scan (*CrysAlis RED*; Oxford Diffraction, 2007)	*k* = −8→10
*T*_min_ = 0.954, *T*_max_ = 0.978	*l* = −23→23
6432 measured reflections	

### Refinement

**Table:**

Refinement on *F*^2^	Primary atom site location: structure-invariant direct methods
Least-squares matrix: full	Secondary atom site location: difference Fourier map
*R*[*F*^2^ > 2σ(*F*^2^)] = 0.041	Hydrogen site location: inferred from neighbouring sites
*wR*(*F*^2^) = 0.129	H-atom parameters constrained
*S* = 1.05	*w* = 1/[σ^2^(*F*_o_^2^) + (0.0833*P*)^2^] where *P* = (*F*_o_^2^ + 2*F*_c_^2^)/3
2495 reflections	(Δ/σ)_max_ < 0.001
202 parameters	Δρ_max_ = 0.22 e Å^−^^3^
0 restraints	Δρ_min_ = −0.17 e Å^−^^3^

### Special details

**Table:**

Geometry. All e.s.d.'s (except the e.s.d. in the dihedral angle between two l.s. planes) are estimated using the full covariance matrix. The cell e.s.d.'s are taken into account individually in the estimation of e.s.d.'s in distances, angles and torsion angles; correlations between e.s.d.'s in cell parameters are only used when they are defined by crystal symmetry. An approximate (isotropic) treatment of cell e.s.d.'s is used for estimating e.s.d.'s involving l.s. planes.
Refinement. Refinement of *F*^2^ against ALL reflections. The weighted *R*-factor *wR* and goodness of fit *S* are based on *F*^2^, conventional *R*-factors *R* are based on *F*, with *F* set to zero for negative *F*^2^. The threshold expression of *F*^2^ > σ(*F*^2^) is used only for calculating *R*-factors(gt) *etc*. and is not relevant to the choice of reflections for refinement. *R*-factors based on *F*^2^ are statistically about twice as large as those based on *F*, and *R*- factors based on ALL data will be even larger.

### Fractional atomic coordinates and isotropic or equivalent isotropic displacement parameters (Å^2^)

**Table:**

	*x*	*y*	*z*	*U*_iso_*/*U*_eq_	Occ. (<1)
O1	0.03590 (18)	0.47993 (15)	0.16546 (7)	0.0582 (4)	
H1O	0.0038	0.5417	0.1934	0.070*	0.36 (3)
O2	0.11482 (19)	0.12327 (15)	0.24445 (7)	0.0600 (4)	
H2O	0.0821	0.0752	0.2769	0.072*	0.64 (3)
O3	0.1437 (2)	0.91245 (18)	−0.08314 (9)	0.0810 (5)	
O4	0.32836 (18)	0.73565 (16)	−0.09586 (7)	0.0624 (4)	
O5	0.57970 (19)	−0.17153 (18)	0.04796 (7)	0.0688 (4)	
O6	0.5900 (2)	−0.35343 (18)	0.12493 (8)	0.0745 (5)	
N1	0.22628 (18)	0.25045 (16)	0.13638 (7)	0.0452 (4)	
H1N	0.1589	0.2915	0.1624	0.054*	
N2	0.2182 (2)	0.79148 (18)	−0.06471 (8)	0.0518 (4)	
N3	0.54401 (19)	−0.22526 (18)	0.10334 (8)	0.0515 (4)	
C1	0.0787 (2)	0.5537 (2)	0.11302 (9)	0.0448 (4)	
C2	0.0151 (2)	0.7055 (2)	0.09352 (10)	0.0496 (4)	
H2A	−0.0587	0.7535	0.1202	0.060*	
C3	0.0605 (2)	0.7798 (2)	0.03718 (9)	0.0480 (4)	
H3A	0.0173	0.8782	0.0253	0.058*	
C4	0.1732 (2)	0.70949 (19)	−0.00398 (9)	0.0440 (4)	
C5	0.2393 (2)	0.56601 (19)	0.01239 (9)	0.0432 (4)	
H5A	0.3139	0.5216	−0.0150	0.052*	
C6	0.1948 (2)	0.48596 (18)	0.07028 (8)	0.0413 (4)	
C7	0.2629 (2)	0.3352 (2)	0.08516 (9)	0.0451 (4)	
H7A	0.3376	0.2956	0.0565	0.054*	
C8	0.2832 (2)	0.09769 (19)	0.15516 (8)	0.0423 (4)	
C9	0.2213 (2)	0.0350 (2)	0.21425 (8)	0.0468 (4)	
C10	0.2737 (3)	−0.1128 (2)	0.23769 (9)	0.0552 (5)	
H10A	0.2348	−0.1551	0.2771	0.066*	
C11	0.3830 (2)	−0.1967 (2)	0.20269 (9)	0.0521 (5)	
H11A	0.4196	−0.2947	0.2187	0.062*	
C12	0.4378 (2)	−0.1335 (2)	0.14340 (8)	0.0443 (4)	
C13	0.3896 (2)	0.01318 (19)	0.11899 (8)	0.0434 (4)	
H13A	0.4281	0.0539	0.0792	0.052*	

### Atomic displacement parameters (Å^2^)

**Table:**

	*U*^11^	*U*^22^	*U*^33^	*U*^12^	*U*^13^	*U*^23^
O1	0.0756 (9)	0.0495 (7)	0.0590 (8)	0.0052 (7)	0.0411 (7)	0.0022 (6)
O2	0.0794 (9)	0.0546 (8)	0.0554 (8)	0.0097 (7)	0.0411 (7)	0.0059 (6)
O3	0.1039 (12)	0.0618 (9)	0.0839 (11)	0.0172 (9)	0.0357 (9)	0.0286 (8)
O4	0.0727 (9)	0.0637 (8)	0.0571 (8)	−0.0062 (7)	0.0302 (7)	0.0013 (7)
O5	0.0802 (10)	0.0740 (10)	0.0606 (8)	0.0124 (8)	0.0375 (7)	−0.0015 (7)
O6	0.0804 (10)	0.0618 (9)	0.0859 (11)	0.0272 (8)	0.0270 (8)	0.0051 (8)
N1	0.0526 (8)	0.0419 (7)	0.0464 (8)	0.0009 (6)	0.0247 (6)	−0.0008 (6)
N2	0.0598 (9)	0.0486 (9)	0.0496 (8)	−0.0094 (7)	0.0163 (7)	−0.0001 (7)
N3	0.0475 (8)	0.0537 (9)	0.0547 (9)	0.0054 (7)	0.0127 (7)	−0.0049 (7)
C1	0.0481 (9)	0.0434 (9)	0.0467 (9)	−0.0053 (8)	0.0193 (7)	−0.0017 (7)
C2	0.0519 (10)	0.0444 (9)	0.0572 (10)	0.0024 (8)	0.0233 (8)	−0.0053 (8)
C3	0.0513 (10)	0.0380 (9)	0.0566 (10)	0.0011 (7)	0.0146 (8)	−0.0003 (8)
C4	0.0470 (9)	0.0397 (9)	0.0469 (9)	−0.0046 (7)	0.0121 (7)	−0.0017 (7)
C5	0.0442 (9)	0.0413 (9)	0.0473 (9)	−0.0035 (7)	0.0171 (7)	−0.0056 (7)
C6	0.0436 (9)	0.0372 (8)	0.0459 (9)	−0.0030 (7)	0.0159 (7)	−0.0041 (7)
C7	0.0480 (9)	0.0462 (9)	0.0459 (9)	−0.0011 (8)	0.0228 (7)	−0.0025 (7)
C8	0.0490 (9)	0.0393 (8)	0.0407 (8)	−0.0009 (7)	0.0139 (7)	0.0008 (7)
C9	0.0552 (10)	0.0489 (10)	0.0405 (8)	0.0009 (8)	0.0207 (8)	−0.0001 (7)
C10	0.0739 (13)	0.0542 (10)	0.0420 (9)	0.0033 (9)	0.0237 (9)	0.0097 (8)
C11	0.0639 (12)	0.0459 (9)	0.0483 (9)	0.0081 (8)	0.0145 (8)	0.0081 (8)
C12	0.0437 (9)	0.0476 (9)	0.0437 (8)	0.0022 (7)	0.0127 (7)	−0.0030 (7)
C13	0.0475 (9)	0.0447 (9)	0.0411 (8)	−0.0049 (7)	0.0167 (7)	0.0017 (7)

### Geometric parameters (Å, °)

**Table:**

O1—C1	1.2749 (19)	C3—C4	1.414 (2)
O1—H1O	0.8200	C3—H3A	0.9300
O2—C9	1.3316 (19)	C4—C5	1.361 (2)
O2—H2O	0.8200	C5—C6	1.393 (2)
O3—N2	1.225 (2)	C5—H5A	0.9300
O4—N2	1.2283 (19)	C6—C7	1.420 (2)
O5—N3	1.2264 (19)	C7—H7A	0.9300
O6—N3	1.216 (2)	C8—C13	1.378 (2)
N1—C7	1.288 (2)	C8—C9	1.405 (2)
N1—C8	1.420 (2)	C9—C10	1.393 (3)
N1—H1N	0.8600	C10—C11	1.378 (2)
N2—C4	1.448 (2)	C10—H10A	0.9300
N3—C12	1.455 (2)	C11—C12	1.384 (2)
C1—C2	1.431 (2)	C11—H11A	0.9300
C1—C6	1.444 (2)	C12—C13	1.381 (2)
C2—C3	1.347 (2)	C13—H13A	0.9300
C2—H2A	0.9300		
			
C1—O1—H1O	109.5	C6—C5—H5A	120.1
C9—O2—H2O	109.5	C5—C6—C7	118.51 (14)
C7—N1—C8	128.10 (14)	C5—C6—C1	120.56 (15)
C7—N1—H1N	116.0	C7—C6—C1	120.92 (14)
C8—N1—H1N	116.0	N1—C7—C6	123.19 (15)
O3—N2—O4	122.95 (15)	N1—C7—H7A	118.4
O3—N2—C4	118.60 (15)	C6—C7—H7A	118.4
O4—N2—C4	118.45 (15)	C13—C8—C9	121.00 (15)
O6—N3—O5	122.63 (16)	C13—C8—N1	124.02 (14)
O6—N3—C12	118.91 (15)	C9—C8—N1	114.98 (14)
O5—N3—C12	118.43 (15)	O2—C9—C10	124.19 (15)
O1—C1—C2	122.32 (15)	O2—C9—C8	116.76 (15)
O1—C1—C6	120.74 (15)	C10—C9—C8	119.05 (15)
C2—C1—C6	116.94 (14)	C11—C10—C9	120.25 (15)
C3—C2—C1	121.08 (15)	C11—C10—H10A	119.9
C3—C2—H2A	119.5	C9—C10—H10A	119.9
C1—C2—H2A	119.5	C10—C11—C12	119.28 (16)
C2—C3—C4	120.53 (16)	C10—C11—H11A	120.4
C2—C3—H3A	119.7	C12—C11—H11A	120.4
C4—C3—H3A	119.7	C13—C12—C11	122.07 (16)
C5—C4—C3	121.09 (15)	C13—C12—N3	118.20 (14)
C5—C4—N2	119.60 (14)	C11—C12—N3	119.68 (16)
C3—C4—N2	119.31 (15)	C8—C13—C12	118.30 (14)
C4—C5—C6	119.79 (15)	C8—C13—H13A	120.8
C4—C5—H5A	120.1	C12—C13—H13A	120.8
			
O1—C1—C2—C3	−178.96 (18)	C7—N1—C8—C13	−0.8 (3)
C6—C1—C2—C3	0.9 (3)	C7—N1—C8—C9	179.85 (17)
C1—C2—C3—C4	−0.3 (3)	C13—C8—C9—O2	−177.90 (15)
C2—C3—C4—C5	−0.4 (3)	N1—C8—C9—O2	1.5 (2)
C2—C3—C4—N2	179.32 (16)	C13—C8—C9—C10	2.3 (3)
O3—N2—C4—C5	172.16 (17)	N1—C8—C9—C10	−178.38 (16)
O4—N2—C4—C5	−7.1 (2)	O2—C9—C10—C11	179.31 (18)
O3—N2—C4—C3	−7.6 (3)	C8—C9—C10—C11	−0.9 (3)
O4—N2—C4—C3	173.17 (15)	C9—C10—C11—C12	−1.0 (3)
C3—C4—C5—C6	0.5 (3)	C10—C11—C12—C13	1.6 (3)
N2—C4—C5—C6	−179.26 (15)	C10—C11—C12—N3	−175.91 (16)
C4—C5—C6—C7	178.68 (15)	O6—N3—C12—C13	178.63 (16)
C4—C5—C6—C1	0.2 (3)	O5—N3—C12—C13	−3.4 (3)
O1—C1—C6—C5	179.03 (16)	O6—N3—C12—C11	−3.7 (3)
C2—C1—C6—C5	−0.8 (2)	O5—N3—C12—C11	174.28 (18)
O1—C1—C6—C7	0.5 (3)	C9—C8—C13—C12	−1.7 (2)
C2—C1—C6—C7	−179.29 (16)	N1—C8—C13—C12	179.01 (16)
C8—N1—C7—C6	178.42 (16)	C11—C12—C13—C8	−0.3 (3)
C5—C6—C7—N1	−178.28 (16)	N3—C12—C13—C8	177.31 (15)
C1—C6—C7—N1	0.2 (3)		

### Hydrogen-bond geometry (Å, °)

**Table:**

*D*—H···*A*	*D*—H	H···*A*	*D*···*A*	*D*—H···*A*
O1—H1O···O2^i^	0.82	1.77	2.5570 (16)	161
O2—H2O···O1^ii^	0.82	1.75	2.5570 (16)	166
N1—H1N···O1	0.86	1.90	2.6001 (19)	138
C3—H3A···O3^iii^	0.93	2.56	3.295 (2)	137
C7—H7A···O4^iv^	0.93	2.67	3.289 (2)	125
C7—H7A···O5^v^	0.93	2.44	3.312 (2)	156
C10—H10A···O4^vi^	0.93	2.53	3.321 (2)	143
C13—H13A···O4^iv^	0.93	2.64	3.195 (2)	119
C13—H13A···O5^v^	0.93	2.63	3.512 (2)	160

Symmetry codes: (i) −*x*, *y*+1/2, −*z*+1/2; (ii) −*x*, *y*−1/2, −*z*+1/2; (iii) −*x*, −*y*+2, −*z*; (iv) −*x*+1, −*y*+1, −*z*; (v) −*x*+1, −*y*, −*z*; (vi) *x*, −*y*+1/2, *z*+1/2.
